# Global warming induced spread of the highest human fascioliasis hyperendemic area

**DOI:** 10.1186/s13071-024-06514-z

**Published:** 2024-10-21

**Authors:** Pablo F. Cuervo, M. Dolores Bargues, Patricio Artigas, Paola Buchon, Rene Angles, Santiago Mas-Coma

**Affiliations:** 1 Departamento de Parasitología, Facultad de Farmacia, Universidad de Valencia, Av. Vicent Andrés Estellés s/n, Burjassot, 46100 Valencia, Spain; 2 CIBER de Enfermedades Infecciosas, Instituto de Salud Carlos III, C/ Monforte de Lemos 3-5. Pabellón 11. Planta 0, 28029 Madrid, Spain; 3 Unidad de Limnología, Instituto de Ecología, Universidad Mayor de San Andrés (UMSA), Campus Calle 27, Cota Cota, La Paz, Bolivia; 4 Cátedra de Parasitología, Facultad de Medicina, Universidad Mayor de San Andrés (UMSA), Av. Saavedra, Miraflores, La Paz, Bolivia

**Keywords:** *Fasciola hepatica*, Lymnaeid snail vector populations, Transmission risk, Forecast indices, Climatic trends, Human hyperendemic area, Northern Bolivian Altiplano

## Abstract

**Background:**

Climate change is driving the occurrence of several infectious diseases. Within a One Health action to complement the ongoing preventive chemotherapy initiative against human fascioliasis in the Northern Bolivian Altiplano hyperendemic area, field surveys showed a geographical expansion of its lymnaeid snail vector. To assess whether climate change underlies this spread of the infection risk area, an in-depth analysis of the long-term evolution of climatic factors relevant for *Fasciola hepatica* development was imperative.

**Methods:**

We used monthly climatic data covering at least a 30-year period and applied two climatic risk indices, the water-budget-based system and the wet–day index, both of verified usefulness for forecasting fascioliasis transmission in this endemic area. To reveal the long-term trends of the climatic factors and forecast indices, we applied procedures of seasonal-trend decomposition based on locally weighed regression and trend analysis on the basis of linear models. To further demonstrate the changes detected, we depicted selected variables in the form of anomalies.

**Results:**

This study revealed a notorious climatic change affecting most of the hyperendemic area, with a strong impact on crucial aspects of the fascioliasis transmission. Trends in maximum and mean temperatures show significant increases throughout the endemic area, while trends in minimum temperatures are more variable. Precipitation annual trends are negative in most of the localities. Trends in climatic risk indices show negative trends at lower altitudes or when farther from the eastern Andean chain. However, monthly and yearly values of climatic risk indices indicate a permanent transmission feasibility in almost every location.

**Conclusions:**

Warmer temperatures have enabled lymnaeids to colonize formerly unsuitable higher altitudes, outside the endemicity area verified in the 1990s. Further, drier conditions might lead to an overexploitation of permanent water collections where lymnaeids inhabit, favoring fascioliasis transmission. Therefore, the present preventive chemotherapy by annual mass treatments is in need to widen the area of implementation. This study emphasizes the convenience for continuous monitoring of nearby zones for quick reaction and appropriate action modification.

**Graphical Abstract:**

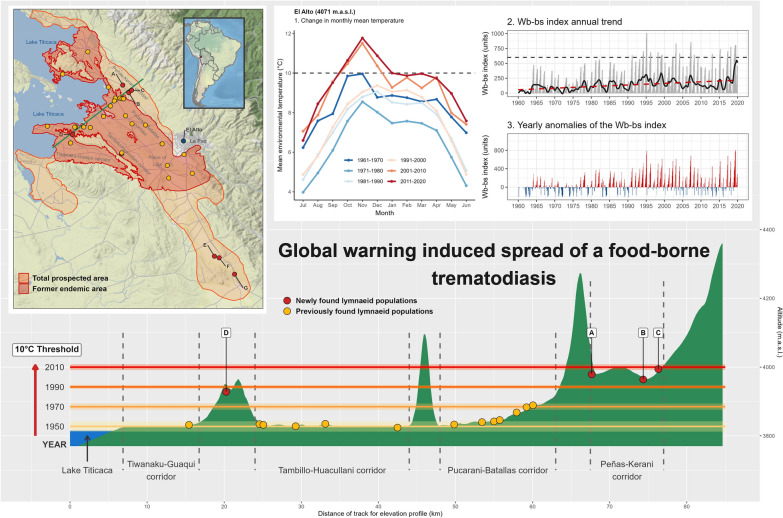

## Background

Human activities and their effects on the climate and environment are causing unprecedented changes in several ecological processes and affecting biodiversity interactions [1]. A growing body of evidence suggests that climate change is already driving the occurrence and transmission of certain diseases [2]. Hence, the World Health Organization (WHO) has claimed to act with urgency on the current climate and health crises [3], emphasizing the need to reinforce appropriate initiatives to mitigate its effects on the transmission of neglected tropical diseases (NTDs) [4].

Vector-borne diseases are particularly susceptible to climate change effects, because arthropod and snail vectors are very sensitive to climate [5]. The strongest climate change impact is observed in zoonotic diseases which show low host specificity at the level of both vector and definitive host, and in which the absence of premunition in the mammal host furnish no buffer effect at the life cycle end. Among helminthiases of these characteristics, fascioliasis has become a standard example since the impact of climate change on trematodiases began to be recognized [6, 7].

Fascioliasis stands out as a significant veterinary concern due to its substantial economic impact on livestock, especially in cattle and sheep farming sectors [8]. From a public health perspective, human fascioliasis has become a growing concern between the 1990s and 2000s [9]. The identification of areas where human fascioliasis is endemic across numerous countries, along with a worldwide increase in the number of reported cases, underscores its increasing global impact [10]. This worrying scenario, combined with its pronounced pathogenicity [11], long-lasting health effects [11, 12], immune system suppression in both acute and chronic phases [13, 14], and its detrimental impact on vulnerable populations in low-income countries [15], led the WHO to categorize fascioliasis as one of the Foodborne Trematodiases included in its priority list of neglected tropical diseases (NTDs) in the WHO NTD Roadmaps for 2020 and 2030 [4, 16]. Further, the WHO stressed the need for an integrated One Health approach to effectively achieve the roadmaps goals [17].

This disease, caused by the two liver fluke species *Fasciola hepatica* and *Fasciola gigantica*, follows a two-host life cycle. Therefore, three of the four phases of their cycle strongly depend on environmental and climatic features [18]:

- egg shedding with mammal feces, egg embryonation in the outer environment, hatching of the miracidium in freshwater and its penetration into an aquatic or amphibious Lymnaeidae snail;
- intramolluscan asexual larval multiplication inside the poikilothermic snail;
- cercarial shedding by the lymnaeid and short swimming phase until transformation into infective immobile metacercarial cyst attached to aquatic vegetation or floating in freshwater.

The fourth phase concerns the definitive host infection, including juvenile migration and final adult stage development in biliary canals and gallbladder of the mammal host. This phase is not directly affected by climate factors, but much influenced by human management and man-guided movements of livestock, and in human fascioliasis by human diet, behavior, and culinary traditions [19].

The aforementioned climatic and anthropogenic influences may modify the local transmission rates of fascioliasis and lead to changes of infection prevalences and intensities in humans and animals. However, the potential impact on the geographical spread of the disease is difficult to assess, and has so far never been reported, because fascioliasis has a worldwide distribution [20]. This is indeed the vector-borne disease presenting the widest latitudinal, longitudinal, and altitudinal distribution known [9]. The assessment of a geographic spread of the fascioliasis transmission risk area could be made during the ongoing wide One Health initiative on the Northern Bolivian human fascioliasis hyperendemic area [21].

In the American continent, *F*. *hepatica* is the only fasciolid present, after its first introduction with livestock imported in the old vessels of the Spanish conquerors 500 years ago, as verified after deep multidisciplinary re-evaluation [10]. The highest public health problems posed by human fascioliasis have been reported from Andean highlands where it is transmitted by the lymnaeid snail species *Galba truncatula*, also imported by the Spanish “conquistadores” [10, 22].

The Northern Altiplano is a human fascioliasis endemic area which is characterized by two aspects which differ from all other human endemic areas in the Andean highlands: (i) it is the endemic area distributed throughout the highest altitudes so far known, and (ii) it is the only Andean endemic area in which fascioliasis is transmitted by only one lymnaeid vector species, i.e., more than one lymnaeid species are involved in other Andean areas, as for instance in Peru [23], Venezuela [24], Chile [25] or Argentina [26].

**Fig. 1:**
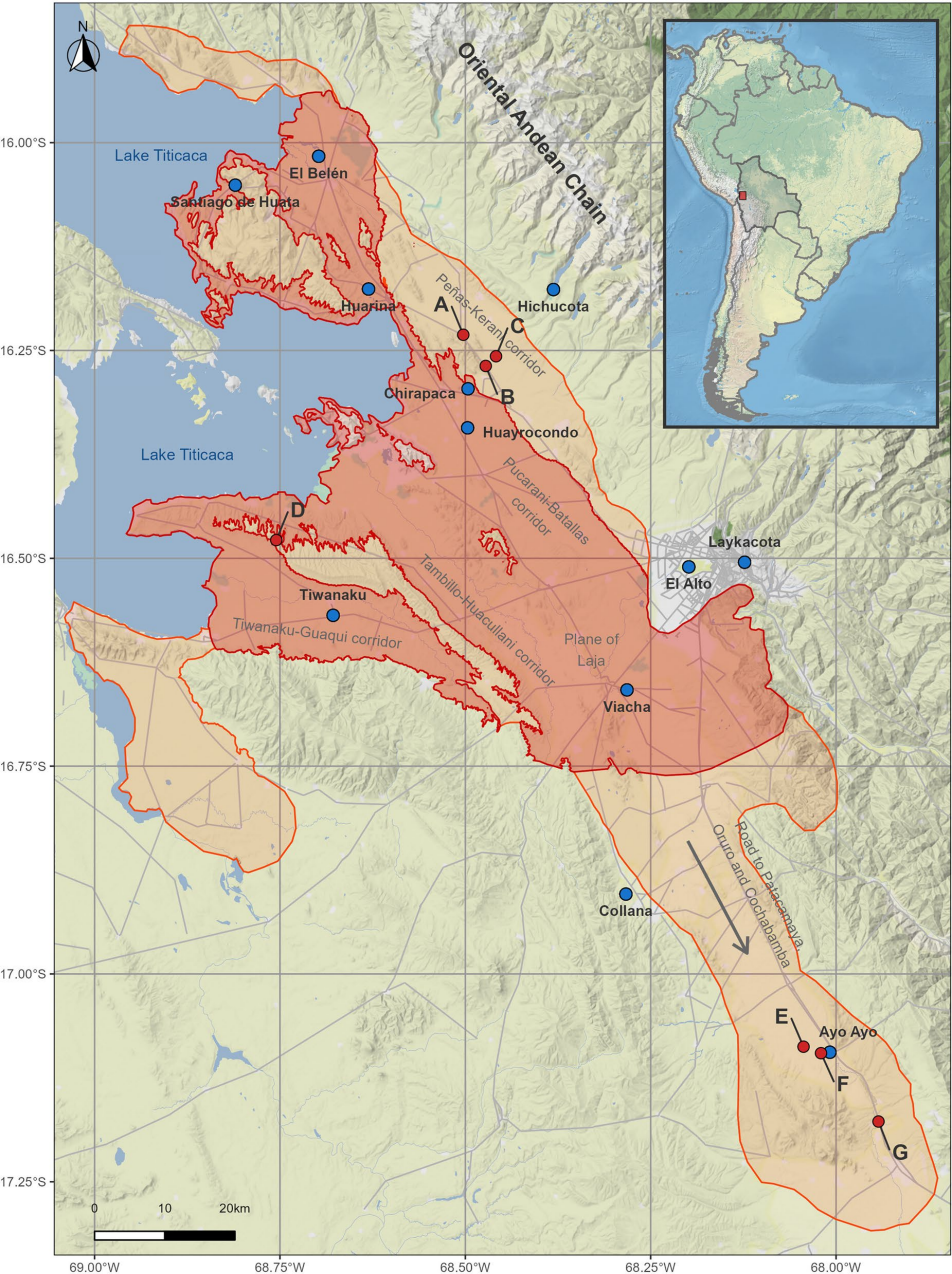
Study area in the Northern Bolivian Altiplano human fascioliasis hyperendemic area. The map shows the meteorological stations included in the study (blue circles), and the locations where populations of lymnaeids has been detected outside the past‑established endemic boundaries (red circles, after [27]). Locations with new-established populations of lymnaeids are listed as: **A** Peñas, **B** San Calixto, **C** Suriquiña, **D** Rosa Pata, **E** Challapata, **F** Ayo Ayo, and **G** Viscachani. Total prospected area, in orange (modified from [15, 27]); and former endemic area defined throughout the 1990’s [29], in red

**Fig. 2:**
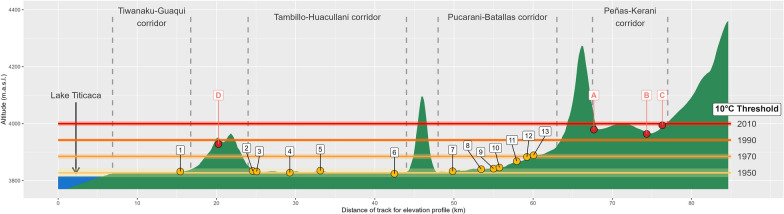
Elevation profile of the human and animal fascioliasis hyperendemic area in the Northern Bolivian Altiplano, depicting the altitudes reached by 10 °C mean annual temperatures in selected decades since 1950. Yellow circles represent locations with presence of lymnaeid populations during the 1990’s surveys, while the red circles represent the four new lymnaeid populations found at higher altitudes during the last surveys (namely, Peñas -A-, San Calixto -B-, Suriquiña -C-, and Rosa Pata -D-). References: 1. Yanarico; 2. Huacullani west; 3. Huacullani north; 4. Lacaya; 5. Quiripujo; 6. Aygachi; 7. Cutusuma; 8. Chijipata Alto; 9. Batallas slaughterhouse; 10. Batallas canal; 11. Río Karawisa; 12. Chirapaca 1; 13. Chirapaca 2 (further details on these locations available in [27])

Recent field surveys throughout this hyperendemic area in Bolivia allowed for the detection of lymnaeid vector populations in localities outside the borders of the endemic area defined throughout the 1992–1997 period [27] (see Fig. 1). Lymnaeid snails could never be found in the freshwater collections in the zones including these new localities, despite having been exhaustively and repeatedly surveyed along all year seasons in this past period (see the total prospected area in Fig. 1). The new localities indicate a wide geographical spread of the infection risk in three different directions. Therefore, representing important repercussions for the ongoing preventive chemotherapy strategy including yearly mass treatment campaigns and corresponding implementation logistics:

- three localities in the Peñas-Kerani corridor (A, B, and C in Fig. 1), indicating an altitudinal northward spread towards the foothills of the eastern Andean chain (Fig. 2);
- two sites in the locality of Rosa Pata in the hill chain separating the two corridors of Tambillo-Huacullani and Tiwanaku-Guaqui (D in Fig. 1), indicating a spread to higher altitude (Fig. 2);
- three localities in the Ayo Ayo-Patacamaya zone (E, F, and G in Fig. 1), indicating a southward spread and increasing remoteness regarding the climate mildering influence of the Lake Titicaca.

All these new localities are situated close to human dwellings, communities, and/or rural schools for children, and interviewed local inhabitants referred to liver fluke infection of their animals. This spread was unexpected, as these areas were formerly deemed unsuitable owing to the low night temperatures. It should be noted that the larval development of *F*. *hepatica* and the growth and reproduction of *G*. *truncatula* are arrested below 10 °C [27, 28].

The purpose of the present study is to assess whether climate change underlies this spread of the infection risk area or it is only the consequence of passive transport of lymnaeid snails when in the mud attached to the hooves of the livestock moved by humans to a priori favorable zone to which they never arrived before. Therefore, we analyzed the long-term evolution of climatic factors relevant for *F*. *hepatica* development throughout the fascioliasis hyperendemic area to assess its putative association.

## Methods

### Study area

The study was focused on the Northern Bolivian Altiplano human fascioliasis hyperendemic area, located between Lake Titicaca and the valley of La Paz city (14–17.5° S, 67.5–71° W) at an altitude ranging between 3800 and 4100 m above sea level (Fig. 1) [29]. This hyperendemic area is where the highest prevalences and intensities of fascioliasis have been reported in humans [29], rising to 72% and 100% prevalence by coprology and serology according to localities, respectively [21]. Children become infected very early in their lives, with more than 3000 eggs per gram of faeces (epg) [30], even reaching up to 8000 epg [31]. Transmission foci are permanent and scattered throughout the hyperendemic area [30, 32]. As aforementioned, this hyperendemic area differs from another Andean human endemic areas owing to its higher altitude and the presence of a single lymnaeid vector species. Particular attention was paid to the areas where geographical spread of lymnaeids has been recently described [27] (see red circles in Fig. 1).

### Climatic data

Monthly climatic data from 12 meteorological stations located in the Northern Bolivian Altiplano were retrieved from the Bolivian *Servicio Nacional de Meteorología e Hidrología* [33]. The climatic data provided covered a standard 30-year climatological reference period in every case but comprised longer periods in most cases. The meteorological stations included in this study are detailed in Table 1, and its geographical location is depicted in Fig. 1.

**Table 1:** Meteorological stations and respective time periods analyzed in the region of the Northern Bolivian Altiplano where human fascioliasis is hyperendemic

Station	Department	Province	Geographical coordinates	Altitude	Time period
El Belén	La Paz	Omasuyos	16º 00′ 59ʺ S—68º 41′ 52ʺ W	3833	1949–2017
Santiago de Huata	La Paz	Omasuyos	16º 03′ 04ʺ S—68º 48′ 37ʺ W	3845	1985–2020
Huarina	La Paz	Omasuyos	16º 10′ 34ʺ S—68º 37′ 50ʺ W	3838	1973–2011
Chirapaca	La Paz	Los Andes	16º 17′ 46ʺ S—68º 29′ 47ʺ W	3870	1991–2020
Huayrocondo	La Paz	Los Andes	16º 20′ 35ʺ S—68º 29′ 49ʺ W	3875	1991–2020
Tiwanaku	La Paz	Ingavi	16º 34′ 07ʺ S—68º 40′ 42ʺ W	3863	1973–2016
Hichucota	La Paz	Los Andes	16º 10′ 36ʺ S—68º 22′ 52ʺ W	4460	1979–2020
El Alto	La Paz	Murillo	16º 30′ 37ʺ S—68º 11′ 55ʺ W	4071	1962–2020
Laykacota	La Paz	Murillo	16º 30′ 17ʺ S—68º 07′ 24ʺ W	3632	1945–2020
Viacha	La Paz	Ingavi	16º 39′ 30ʺ S—68º 16′ 55ʺ W	3850	1965–2015
Collana	La Paz	Aroma	16º 54′ 01ʺ S—68º 16′ 54ʺ W	4500	1973–2020
Ayo Ayo	La Paz	Aroma	17º 05′ 39ʺ S—68º 00′ 30ʺ W	3888	1958–2020

Predominant climatic conditions and seasonality were estimated with respect to the climate period from 1981 to 2020 for most meteorological stations. In the few cases where this information was not available (Chirapaca and Huayrocondo), the climate period analyzed was from 1991 to 2020. The monthly data analyzed were mean environmental temperature (MET), mean maximum temperature (MMT), mean minimum temperature (MmT), extreme maximum temperature (EMT), and extreme minimum temperature (EmT), all in °C, precipitation (Pt), maximum precipitation (MP), total potential evapotranspiration (PET), all in mm, number of days with precipitation (DP) and number of days with frost (DF). In addition, yearly precipitation (YP) considered the mean precipitation cumulated throughout an entire year.

### Climatic forecast indices

The incidence of fascioliasis infection in the definitive host has been related to air temperature, rainfall, and/or potential evapotranspiration [7]. Climatic forecast indices calculate growing degree-days (GDDs) above a threshold temperature and use rainfall and evapotranspiration data, whereas its output is a numerical value that indicates fascioliasis risk. Optimal climatic conditions allow for rapid development from egg to encysted metacercaria, logically conferring a high risk of infection. This approach assumes the mandatory presence of the snail host and includes all non-parasitic stages into one model. Its main limitation is that these forecast indices are empirical in nature and exploit correlations between historical data. Therefore, they are argued to have limited ability to assess future risk and guide interventions under changing conditions [34]. However, its relative simplicity points them as potentially accessible to the health sector and any governmental agency, especially from developing countries, so as to identify when and where the risk of transmission is possible, narrowing the area under risk. Its usefulness has been proven in some countries in Africa [35, 36], America [18, 37, 38], Asia [39], and Oceania [40].

The two most useful indices have proved to be the Wet Day index or Mt index [41] and its later improved versions [42, 43], and the Water budget-based system index or Wb-bs index [44] and its later modified version for large-scale regional use [35].

The Mt index is expressed by the equation:

$$\text{Mt }=\text{ n }(\text{R}-\text{PET }+ 125)/25$$

where *n* is the number of rain days, *R* is the rainfall in mm, and *PET* is the potential evapotranspiration in mm. For the calculation of this index, the only months considered are those in which the MET is ≥ 10 °C, since this temperature is considered the lower threshold temperature for the development of fascioliasis by *F*. *hepatica* [41, 45]. 

The Wb-bs index, adapted for large scale regional application using monthly climatic data, is expressed as:

$$\begin{aligned} {\text{Wb}} - {\text{bs }} & = \,\left( {{\text{GDD }} \times {\text{ days in month}}} \right),{\text{ if }}\left[ {{\text{R }} - { }\left( {{\text{PET x }}0.8} \right)} \right]{ } > 0, \\ & + { }\left( {\text{GDD x n}} \right){ } \times { }\left[ {\left( {{\text{R }} - {\text{ PET}}} \right)/25} \right],{\text{ if }}\left( {{\text{R }} - {\text{ PET}}} \right){ } > 0 \\ \end{aligned}$$

where *R* is the rainfall, *PET* the potential evapotranspiration, *n* the monthly number of days with surplus rainfall (> 1 mm), and *GDD* the growing degree-days calculated as the monthly MET-10 °C [46]. In the first part of the formula, subtracting the factor (*PET* × 0.8) from rainfall (*R*) is assumed to be equivalent to counting monthly GDD if moisture storage is present in the top 2.5 cm layer of a soil water budget model. The second part counts GDD if monthly surplus water is present owing to rainfall events [35, 44].

After introducing modifications for high altitude and low latitude, these two climatic forecast indices have been previously applied in the Bolivian Altiplano [18]. Climate diagrams furnished appropriate results on the duration of the wet and dry seasons only after introducing a modification concerning aridity influence [7, 47]. The Mt and Wb-bs forecast indices should, therefore, be accordingly modified, as successfully proved when previously applied to this human fascioliasis hyperendemic area of the Bolivian Altiplano [18]. Potential evapotranspiration (PET) is replaced by Schreiber’s aridity index *r* [47] (named from now on as *AI*), and calculated as follows:

$${\text{AI }} = { }\left( {2{\text{t}}_{{\text{k}}} { } + { }0.03{\text{t}}^{{\text{k}}}_{2} } \right){ } \times { }\left( {{\text{S}}/12} \right)$$

where *t*_*k*_ is the corrected mean monthly temperature (which is increased by an altitude factor), and *S* is the mean monthly daylight in hours (which becomes increasingly noticeable at higher latitudes). Additionally, as the MmT (often corresponding to night-time temperatures) reached in a large part of the study area causes the MET to fall below of 10 °C for much of the year, the calculations were modified to give relevance to the MMT [38], which exceeds the minimum temperature required for the start of activity of the lymnaeid host and free-living stages of *F*. *hepatica* during long periods of the year.

Summarizing, the two indices were calculated according to the formulae proposed for high altitudes in tropical or subtropical areas [18]:

$${\text{Mt }} = {\text{ n }}\left( {{\text{R }}{-}{\text{ AI }} + { }125} \right)/25,$$

$$\text{considering only those months in which }[(\text{MET }+\text{ MMT})/2]\text{ is }\ge 10^\circ \text{C}$$

$$\begin{aligned} {\text{Wb}} - {\text{bs }} & = \,\left( {{\text{GDD }} \times {\text{ days in month}}} \right),{\text{ if }}\left[ {{\text{R }} - { }\left( {{\text{AI x }}0.8} \right)} \right]{ } > 0,{ } \\ & + { }\left( {{\text{GDD }} \times {\text{ n}}} \right){ } \times { }\left[ {\left( {{\text{R }} - {\text{ AI}}} \right)/25} \right],{\text{ if }}\left( {{\text{R }} - {\text{ AI}}} \right){ } > 0 \\ \end{aligned}$$

where *AI* is the aridity index and *GDD* = [(MET + MMT)/2]—10, considering only those months in which [(MET + MMT)/2] is ≥ 10 °C.

The months with a value for Mt equal to or higher than a critical value are considered potential high-risk periods for the incidence of the disease. Mt values sufficient to support transmission have been considered as ≥ 100 in UK, 80 in France [42, 43], and as low as 55–60 in Pakistan [39].

We analyzed the Wb-bs index on the basis of accumulative values in a continuous way when different from 0. Risk values conventionally established and used by several authors, are: 600 = no risk; 601–1500 = low risk; 1500–3000 = moderate risk; and 3000 = high risk [18, 35, 36, 38, 39, 46].

### Analysis of the long-term variation of climatic factors and forecast indices

The trend analysis was performed considering the entire dataset, covering more than 30 years in most cases (see Table 1 for the period analysed in each case). Regarding the influence of the climate change, change detection methods are often not capable of detecting changes within time series that are heavily influenced by seasonal climatic variations [48]. To reveal potentially significant trends, we analyzed the impact of the climate change over climatic factors and forecast indices with a seasonal-trend decomposition procedure (STL) based on locally weighed regression (Loess). This procedure, performed with the R packages “forecast” and “zoo” [49, 50], decomposes the time series into trend, seasonal and remainder components [51]. After the decomposition process, we performed linear models to analyze the trend component of each variable. The trend component was considered as the response, whereas “time” was included as explanatory variable to account for the long-term variation in time-series data (trend ~ time). The STL procedure and linear models were performed with the entire dataset (annually) and with the four seasonal subsets: winter [June-July–August (JJA)], spring [September–October-November (SON)], summer [December-January–February (DJF)], and autumn [March–April-May (MAM)]. Results were considered statistically significant when *p-value* < 0.05. To depict the significant trends detected in the area and compare between locations, we expressed the magnitude of change per decade.

In addition, to further demonstrate the changes observed, selected variables were depicted in the form of “anomalies,” which are the departure of the observed value from a reference value or long-term average for the particular month given by a base period [52]. Wherever possible, the 30-year reference period was set to 1961–1990, as recommended by the World Meteorological Organization (WMO) for the computation and tracking of global climate anomalies [53]. A positive anomaly indicates that the observed variable was of greater magnitude than the reference value, while a negative anomaly indicates that the observed variable was of lesser magnitude than the reference value.

### Spatial and statistical analyses

All the aforementioned computations and statistical analyses were performed with R Statistical Software 4.1.2 (“R: A language and environment for statistical computing,” www.r-project.org), and RStudio 2022.02.3.492 (‘RStudio: Integrated development environment for R’, http://www.rstudio.com/).

## Results

Although we analyzed data from meteorological stations located throughout the entire hyperendemic area (Fig. 1), we paid particular attention to those related with the areas where lymnaeid snails have recently spread to. For instance, the meteorological stations of Chirapaca (3870 m.a.s.l.) and El Alto (4071 m.a.s.l.), are altitudinally related to the newly found lymnaeid populations in the Peñas-Kerani corridor (A, B and C in Fig. 1) and those in the hill chain separating the Tambillo-Huacullani and the Tiwanaku-Guaqui corridors (D in Fig. 1), which present altitudes between 3965 and 4001 m.a.s.l. While the meteorological station of Ayo Ayo (3888 m.a.s.l.) is geographically close to the lymnaeid populations recently found in the Ayo Ayo-Patacamaya zone (E, F, and G in Fig. 1).

**Fig. 3:**
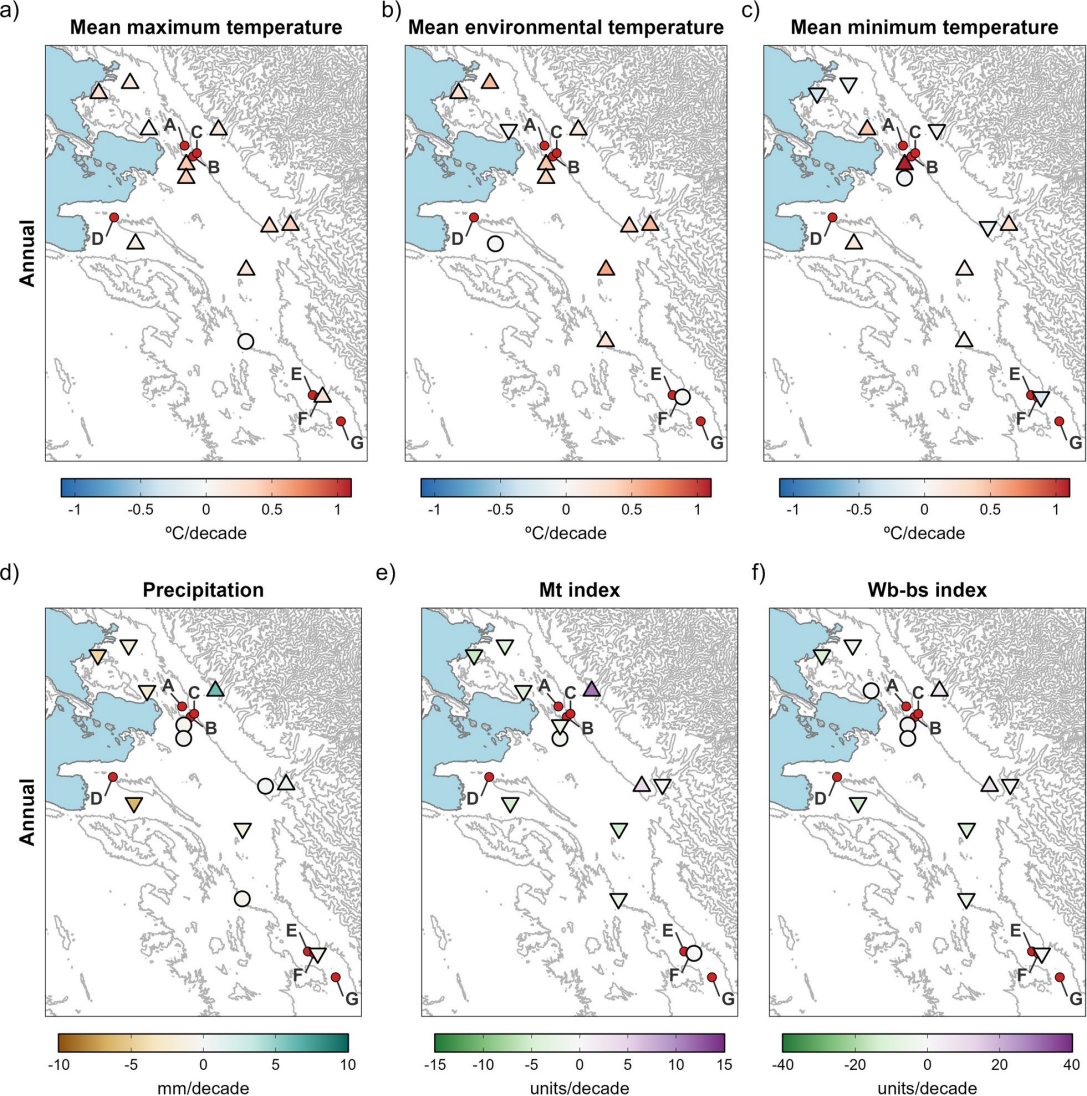
Spatial distribution of annual trends for mean maximum temperature (MMT), mean environmental temperature (MET), mean minimum temperature (MmT), precipitation (Pt), Mt index and Wb-bs index. The gradient of colours denotes the intensity of change. Stations with trends significant at the 0.05 level are marked with an upward or downward triangle to denote positive and negative trends, respectively. Circles depict non-significant trends. Locations with new-established populations of lymnaeids are listed as: (**A**) Peñas, (**B**) San Calixto, (**C**) Suriquiña, (**D**) Rosa Pata, (**E**) Challapata, (**F**) Ayo Ayo, and (**G**) Viscachani. Lake Titicaca is shown as light blue filled area and the elevation as grey contour lines

**Fig. 4:**
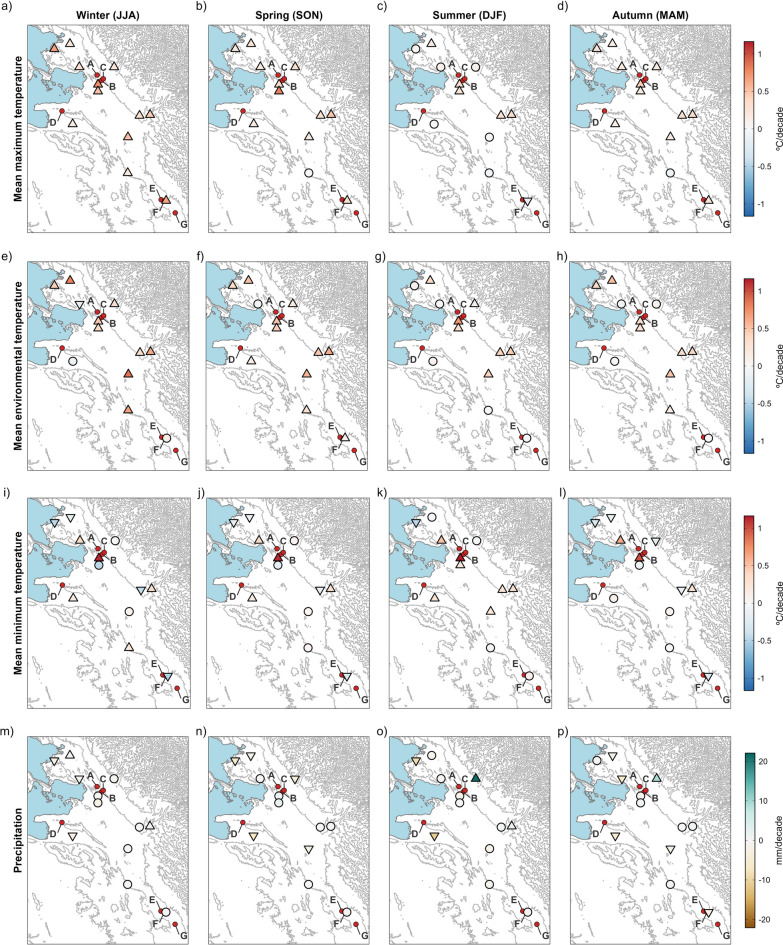
Spatial distribution of seasonal trends for mean maximum temperature (MMT, a–d), mean environmental temperature (MET, e–h), mean minimum temperature (MmT, i–l) and precipitation (Pt, m–p). The gradient of colours denotes the intensity of change. Stations with trends significant at the 0.05 level are marked with an upward or downward triangle to denote positive and negative trends, respectively. Circles depict non-significant trends. Locations with newestablished populations of lymnaeids are listed as: (**A**) Peñas, (**B**) San Calixto, (**C**) Suriquiña, (**D**) Rosa Pata, (**E**) Challapata, (**F**) Ayo Ayo, and (**G**) Viscachani. Lake Titicaca is shown as light blue filled area and the elevation as grey contour lines

**Fig. 5:**
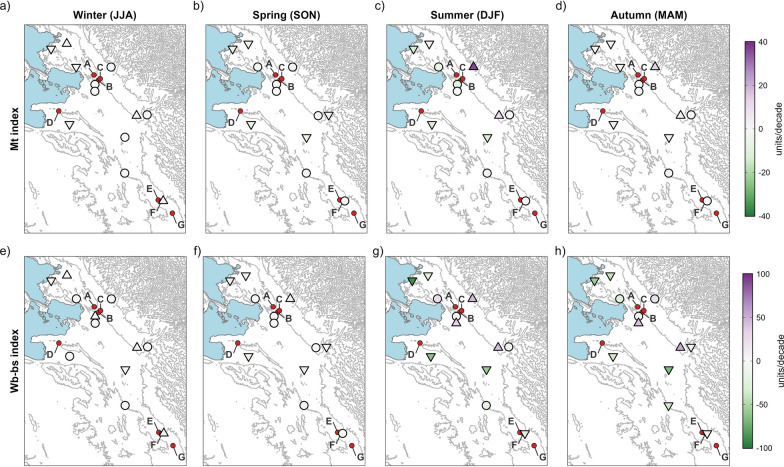
Spatial distribution of seasonal trends for climatic forecast indices (Mt index, a-d; wb-bs index, e-h). The gradient of colours denotes the intensity of change. Stations with trends significant at the 0.05 level are marked with an upward or downward triangle to denote positive and negative trends, respectively. Circles depict non-significant trends. Locations with newestablished populations of lymnaeids are listed as: (**A**) Peñas, (**B**) San Calixto, (**C**) Suriquiña, (**D**) Rosa Pata, (**E**) Challapata, (**F**) Ayo Ayo, and (**G**) Viscachani. Lake Titicaca is shown as light blue filled area and the elevation as grey contour lines

Our results illustrate a significant major climatic change affecting most of the hyperendemic area, with a strong impact on crucial aspects of the fascioliasis transmission. Trends in maximum and mean temperatures are spatially consistent and show significant increases in most of the stations through all seasons (Figs. 3a, b, and 4a–h). Trends in minimum temperatures are more variable, although showing significant negative trends in some localities (Figs. 3c and 4i–l). Annual trends in precipitation are negative in most of the hyperendemic region (Fig. 3d), although seasonal trends are highly variable and non-significant in most of the localities (Fig. 4m–p). Trends in climatic risk indices are spatially consistent, significant in most cases, and show negative trends at lower altitudes or when farther from the eastern Andean chain (Fig. 3e, f and 5).

**Fig. 6:**
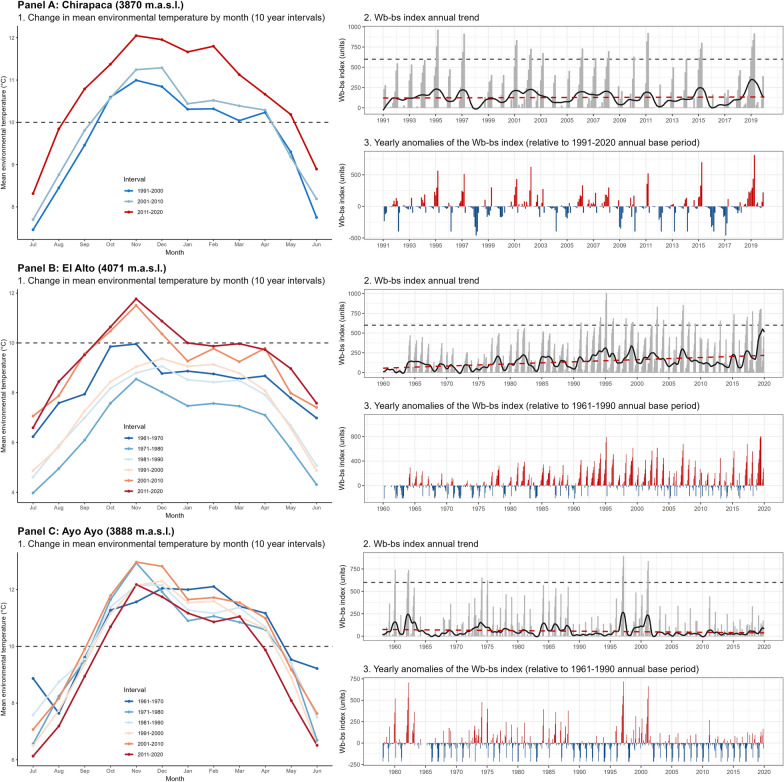
Annual change in mean environmental temperature and Wb-bs index in three selected meteorological locations (Chirapaca, El Alto, and Ayo Ayo) for the analysis of the geographical spread of the fascioliasis transmission area. In each panel, the first figure represents the mean monthly change of mean environmental temperature by intervals of 10 years (the slashed grey line depicts the 10 °C threshold required for the life cycle of *Fasciola hepatica* to progress). The second picture shows the Wb-bs index annual trend, represented by the observed values (grey columns), the observed trend obtained from the seasonal-trend decomposition procedure (black line), and the regression line (red-slashed line) (the slashed grey line depicts the value of 600, which indicates risk of transmission). The third picture shows the Wb-bs index in the form of “anomaly,” which is the difference between the current value and an average value for the particular month given by a base period (positive anomalies are in red, and negative anomalies in blue)

*Northward spread* At the Chirapaca meteorological station, located at an altitude ~ 100 m below that of the lymnaeid populations in the Peñas-Kerani corridor and Rosa Pata (A, B, C, and D in Fig. 1), the recorded maximum and mean temperatures increased ~ 0.45 °C/decade (Fig. 3a, b), while minimum temperatures surpassed an increase of 1 °C /decade (Fig. 3c). The positive trend in mean temperature was stronger during summer (Fig. 4g). Monthly mean temperatures during the 1991–2010 period barely surpassed the 10 °C minimum threshold of *F*. *hepatica* development, but during the subsequent 2011–2020 period monthly mean temperatures were well above 10 °C from August to May (Fig. 6, Panel A1). Moreover, the window without major frosts widened from three months (January to March) to five (November to March) (data not shown). The trends of the climatic risk indices were not significant throughout most of year (Figs. 5 and 6, Panel A2), which appears reflected on consistent anomalies during the 1991–2020 period analyzed (Fig. 6, Panel A3).

*Altitudinal spread* At the meteorological station in El Alto, located ~ 100 m above the altitudes of the aforementioned lymnaeid populations (A, B, C, and D in Fig. 1), mean temperatures were below 10 °C throughout the entire year until de 2000s (Fig. 6, Panel B1). A significant increasing trend of 0.41 °C /decade occurred during the 1962–2020 period (Fig. 3b), including a most notorious 0.48 °C /decade increase in spring, and led mean temperatures to surpass the 10 °C threshold during the last two decades (Fig. 6, Panel B1). The Wb-bs climatic risk index increased significantly during this six-decade period of 1962–2020 (Fig. 3f), including values compatible with fascioliasis transmission since 1990 (Fig. 6, Panel B2), and evinced in the increasing magnitude of the positive anomalies (Fig. 6, Panel B3).

*Southward spread* Regarding the lymnaeids populations in the Ayo Ayo-Patacamaya zone (E, F and G in Fig. 1), at the Ayo Ayo meteorological station, maximum temperatures increased whereas minimum temperatures decreased. This results in non-significant trends in mean environmental temperatures (Figs. 3a-c, 4a-i). In this zone, mean environmental temperature keeps above 10 °C from September to April (Fig. 6, Panel C1). The Wb-bs index decreased significantly (Fig. 6, Panel C2) and appears reflected in decreasing positive anomalies during the period analyzed (Fig. 6, Panel C3), although transmission values are reached occasionally.

## Discussion

The impact of climate change on fascioliasis has already been observed in a number of endemic areas, including developed countries [54, 55], low-income countries [56], and also analyses of general future trends [57]. However, all these studies focused on fascioliasis of livestock. Although an impact of climate change on human fascioliasis endemic areas was expected to occur [6, 7], there is so far only one climate change study dealing on human fascioliasis, namely in the Punjab province of Pakistan [39]. This study was the first to demonstrate a link between climate change and human fascioliasis emergence, highlighting the synergistic effects of the overlap of climate change and global change on fascioliasis transmission by transforming a mono-seasonal transmission pattern into a bi-seasonal one, with a yearly peak linked to increasing monsoon rainfall and another yearly peak linked to irrigation management [39]. Nevertheless, all these studies concern alterations of prevalences in animals or humans caused by modifications of climatic factors in endemic areas. None refer to a climate-related expansion of the transmission area.

The detailed seasonal-trend decomposition procedure applied to our analyses allowed to evidence a remarkable climatic change in the human fascioliasis hyperendemic area of the Northern Bolivian Altiplano. This change proves to be variable at a local scale. This fits within the general warming noted to affect the Peruvian and Bolivian Altiplanos [58–60]. With a few exceptions, maximum, and mean temperatures had a clear increase in the entire hyperendemic area, with warming trends generally between 0.2 and 0.5 °C per decade. Minimum temperatures present an irregular pattern depending on the geographical location within the area, which has been attributed to local factors [59] and reported elsewhere [61]. Trends of minimum temperature are generally lower in magnitude compared with trends in maximum temperature and have been attributed to an elevation-warming dependency which is weakened for minimum temperature trends [62].

It should be considered that temperature is a key factor for fascioliasis transmission. Indeed, the minimum temperature thresholds are crucial for the development of both the fasciolid species and the lymnaeid vector species. However, the way by which the minimum temperature influences differs one another. Results of previous studies performed in this endemic area should be considered: (i) at the extreme altitude of the Altiplano, temperature varies very pronouncedly between day and night, so that the negative impact of the very low night temperatures are counteracted by the relatively high mid-day temperatures [18], this counteracting allowing for the fluke larval stages to survive; (ii) the temperature inside the water of the freshwater transmission foci of the Altiplano does not decrease as much as the environment temperature, so that even when the latter reaches temperatures close to 0 °C the temperature of such waters keeps at a level not killing the fasciolid larval stages [27]; (iii) experimental infection studies performed at 22 ºC/5 °C day/night temperature demonstrated that *F*. *hepatica* from Altiplanic sheep and cattle is able to follow the whole complete larval development inside *G*. *truncatula* from the Altiplano up to finally produce and shed fully viable cercariae [63]; (iv) the lymnaeid vector *G*. *truncatula*, however, is pronouncedly influenced by the environmental temperature, because this poikilothermic snail has a marked amphibious behavior, i.e., follows a daily activity on mud out of water. Hence, at the extreme conditions of the Bolivian Altiplano, it is for the lymnaeid snail to define the outer altitudinal borders. The definite impact of the minimum temperature in mountainous highlands on free living organisms is well known and easily visible, e.g., when observing the clear and precise altitudinal tree lines of the forests in valleys or the maximum altitudinal distributions of poikilothermic invertebrates. Differences in altitudinal distribution of different lymnaeid species have been already observed in the Cajamarca valley endemic area [23].

Precipitation also varies spatially within the hyperendemic area, with decreasing trends in most of the localities. Although there is no previous study specifically dealing with the Northern Bolivian Altiplano, results found in neighboring zones are diverse: a precipitation decrease for the annual total and during the main rainy season at the Peru/Bolivia border [64]; a slight decrease of precipitation in the southern Peruvian Altiplano (located to the west of the Northern Bolivian Altiplano) [65]; an increase in the intensity of rainfall extremes in the Peruvian Titicaca [66], but a non-significant negative trend in total precipitation in the same area reported later [61]; and significant increases in annual precipitation in the northern part of the Desaguadero-Poopó system, located to the southwest of the Northern Bolivian Altiplano [67]. Indeed, non-significant trends in monthly and annual precipitation are the most prevalent across the country, which highlights that natural precipitation variability is the dominant pattern [68].

In our study, the main climatic factors analyzed are those which are included in the formula of the forecast indices because of their verified importance in *F*. *hepatica* transmission. Other climate factors of additional potential influence such as humidity, evapotranspiration, sunshine hours, etc., of this Bolivian Altiplano endemic area were already analyzed previously [18, 69], similarly as it was for other environmental factors such as soil compaction, land use, freshwater collections, livestock management, etc. [15, 27, 29, 32].

Concerning the forecast indices, the long-term pattern shows a general decline, which might result from the general increment of temperature, a consequent increase of evapotranspiration, and the reduction of precipitation in some places. However, the risk of transmission continues to be high in most of the hyperendemic area, as transmission values are easily reached most of the years. Indeed, when analyzing the maximum monthly value and mean yearly values accumulated during an entire year, transmission values are reached, and nearly duplicated, in almost every location, indicating that transmission is feasible through the year.

The warming process at the high altitudes of the Bolivian Altiplano should have a direct impact on the three aforementioned liver fluke cycle phases dependent on environmental features. A general increase of environmental temperatures above 9–10 °C will: (i) affect snail vector population dynamics [32], (ii) enable and shorten the period of egg embryonation, both of *F*. *hepatica* and *G*. *truncatula* [32, 70, 71], (iii) enhance the development and maturation of the intramolluscan larval stages [72], and (iv) stimulate cercarial emergence [45]. It must be considered that fascioliasis transmission in this high-altitude area does not exclusively rely on precipitation, but mainly on the availability of permanent water sources [29, 32].

The finding of new lymnaeid populations outside of the previously known boundaries of the hyperendemic area, in the northern corridor of Peñas-Kerani (A, B, and C in Fig. 1) and in the hill chain separating the Tambillo-Huacullani and the Tiwanaku-Guaqui corridors (D in Fig. 1), can thus be linked to the warming process herein described, which in turn appears enhanced at higher altitudes [73]. Our results show that, in the last 30 years, mean and maximum temperatures increased more than 1 °C in the area between 3870 and 4070 m.a.s.l. (between 1.5 and 2 °C considering only spring and summer). This significant temperature rise results in current diurnal temperatures well above 10 °C for at least 3 months (Fig. 6, Panels A1 and B1), enabling environmental conditions compatible with the reproduction and long-term establishment of new lymnaeid populations. Furthermore, the detailed analysis of the annual trends of the Wb-bs forecast index (Fig. 6, Panels B2 and B3) indicates an increasing risk of fascioliasis transmission in these higher areas since the mid-1990s, which was previously deemed impossible.

The situation in the Ayo-Patacamaya zone appears linked to an increase of the maximum temperatures in spite of a decrease of the minimum temperatures. Indeed, the temperatures during daily hours allow the intra-molluscan larval development of *F*. *hepatica*, counteracting the low nighty air temperatures, which however does not lead to such a temperature decrease of water in the transmission foci [32]. *Galba truncatula* snails are known to endure long cold winters, and seem unaffected by low temperatures itself [70, 74]. Nevertheless, when exposed to temperatures below 10 °C, snail growth is almost totally inhibited and reproduction is supressed [28, 74]. Therefore, the long-term survival of this species is jeopardized in environments where temperatures are below 10 °C during most of the day and throughout the entire year, as occurs in the higher zones of the Bolivian Altiplano. Assisted by passive dispersal, lymnaeid snails might reach these higher grounds, but under such conditions they will not be able to reproduce and thus maintain long-term populations. Ways of the arrival of the lymnaeid vectors to the new localities by means of passive transport by human-guided movements of domestic animals have been reported in the Altiplano, mainly concerning cattle and donkeys [27, 75].

Although climate change has been repeatedly evoked as a phenomenon potentially leading to the geographical spread of fascioliasis, no specific study has so far been able to prove such a possibility. Indeed, a recent countrywide multidisciplinary study conducted in Vietnam to assess the spread of this disease between 1995 and 2019 has demonstrated that climate change was not involved in the geographical expansion of the fasciolids and their snail vector in this climatologically heterogeneous country [76]. In the Bolivian Altiplano, results of several field and experimental studies identified global warming as the potential major cause of fascioliasis spread and helped discard other potential confounders: (i) our recent field monitoring demonstrated active populations of *G*. *truncatula* in the endemic out-border localities originally detected [27], thus confirming their adaptation to the new localities; (ii) presence of livestock facilitating fascioliasis transmission and their closeness to villages, human dwellings, and rural schools; (iii) human infection has been detected in the Peñas-Kerani corridor and Rosa Pata, and cattle infection in the Ayo Ayo-Patacamaya zone, by Bolivian health officers; (iv) the three zones of expansion have therefore already been included within the annual mass treatment campaigns; (v) the capacity of Altiplanic *F*. *hepatica* to successfully infect and give rise to the shedding of viable cercariae in Altiplanic *G*. *truncatula* in experiments at 22 °C/5 °C day/night temperature further prove the viability of fascioliasis in such extreme localities [63]. These findings, and the clear spatial overlap with the changes in climatic factors and climatic forecast indices presented in this study indicate that global warming may be the major cause of the geographical spread of the disease. Indeed, the only confounder about a snail vector passive transport by livestock movements may be discarded, as livestock was widely present and moved through in the three spreading zones already in the 1980s and 1990s.

Furthermore, drier conditions might result in an overexploitation of the remnant sources of water, favoring fascioliasis transmission, as recently described for arid areas in Argentina [26]. In the Bolivian Altiplano, this may favor fascioliasis transmission in this area where the high-altitude evapotranspiration leads lymnaeids to inhabit permanent water collections [29, 32] and rural Aymara inhabitants traditionally rely on natural water for drinking and food preparation [15].

## Conclusions

This temperature-related enhancement of the life cycle of *F*. *hepatica* and the increasing dryness leading to the concentration of freshwater sources might have epidemiological repercussions, boosting the infection and reinfection of the Altiplanic inhabitants. Hence, the present preventive chemotherapy by annual mass treatments is therefore in need to widen the area of implementation. 

This is the in-depth climate change study ever performed in a human fascioliasis endemic area and emphasizes the convenience for continuous monitoring of nearby zones for quick reaction and appropriate action modification. The availability of the widest, long-term, multidisciplinary baseline knowledge on this highly complex disease in a given human endemic area enabled it.

## Data Availability

The data and scripts that support the findings of this study are available from the corresponding author upon reasonable request.
